# Supplementary material for: Quinoa for the Brazilian Cerrado: Agronomic Characteristics of Elite Genotypes under Different Water Regimes
Source: Plants (Basel). 2021 Aug 2;10(8):1591. doi: 10.3390/plants10081591 (PMC8401838; doi:10.3390/plants10081591)
Supplement: Supplementary file 1 [file plants-10-01591-s001.zip › Table S1.pdf]

Table S1. Chlorophyll *a, b, a/b*e *a+b* measured in four quinoa genotypes independent of water regime (150, 247, 389 and 480 mm).

Genotype	Chlorophyll *a*	Chlorophyll *b*	Chlorophyll *a/b*	Chlorophyll *a+b*
CPAC4	44.1b	14.4a	3.13a	59.1bc
CPAC11	43.7c	16.5a	3.28a	57.4c
BRS Piabiru	45.0ab	16.5a	2.90a	61.4b
CPAC19	45.4a	18.9a	2.27b	66.0a

Means followed by the same lowercase letter (column) or uppercase letter (line), do not differ according to the Tukey test at 5% probability.
