# Supplementary material for: Quinoa for the Brazilian Cerrado: Agronomic Characteristics of Elite Genotypes under Different Water Regimes
Source: Plants (Basel). 2021 Aug 2;10(8):1591. doi: 10.3390/plants10081591 (PMC8401838; doi:10.3390/plants10081591)
Supplement: Supplementary file 1 [file plants-10-01591-s001.zip › Table S2.pdf]

Table S2. Proline content (µ mol g^{-1} FM) in leaves of four quinoa genotypes (CPAC4, CPAC 9, CPAC 11 e BRS Piabiru Precoce) under four water regimes (150, 247, 389, 480 mm).

	Water Regime (mm)			
**Genotypes**	**150**	**247**	**389**	**480**
CPAC4	0.09bA	0.07aAB	0.06aB	0.07abAB
CPAC 11	0.13aA	0.07aB	0.06aC	0.05bC
Piabiru	0.11abA	0.08aAB	0.07aB	0.07abB
CPAC 19	0.11abA	0.09aA	0.09aA	0.09aA

Means followed by the same lowercase letter (column) or uppercase letter (line), do not differ according to the Tukey test at 5% probability.
