# Supplementary figures and images for: Quinoa for the Brazilian Cerrado: Agronomic Characteristics of Elite Genotypes under Different Water Regimes
Source: Plants (Basel). 2021 Aug 2;10(8):1591. doi: 10.3390/plants10081591 (PMC8401838; doi:10.3390/plants10081591)

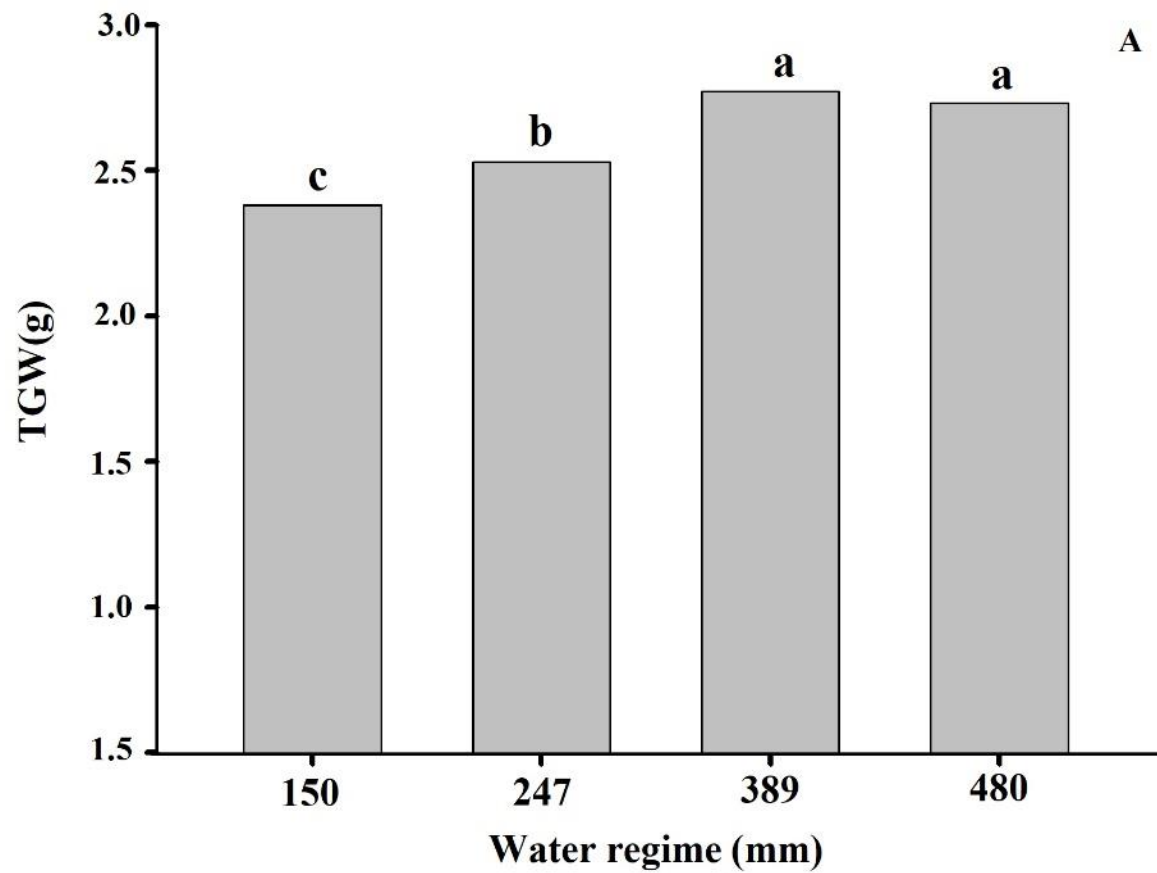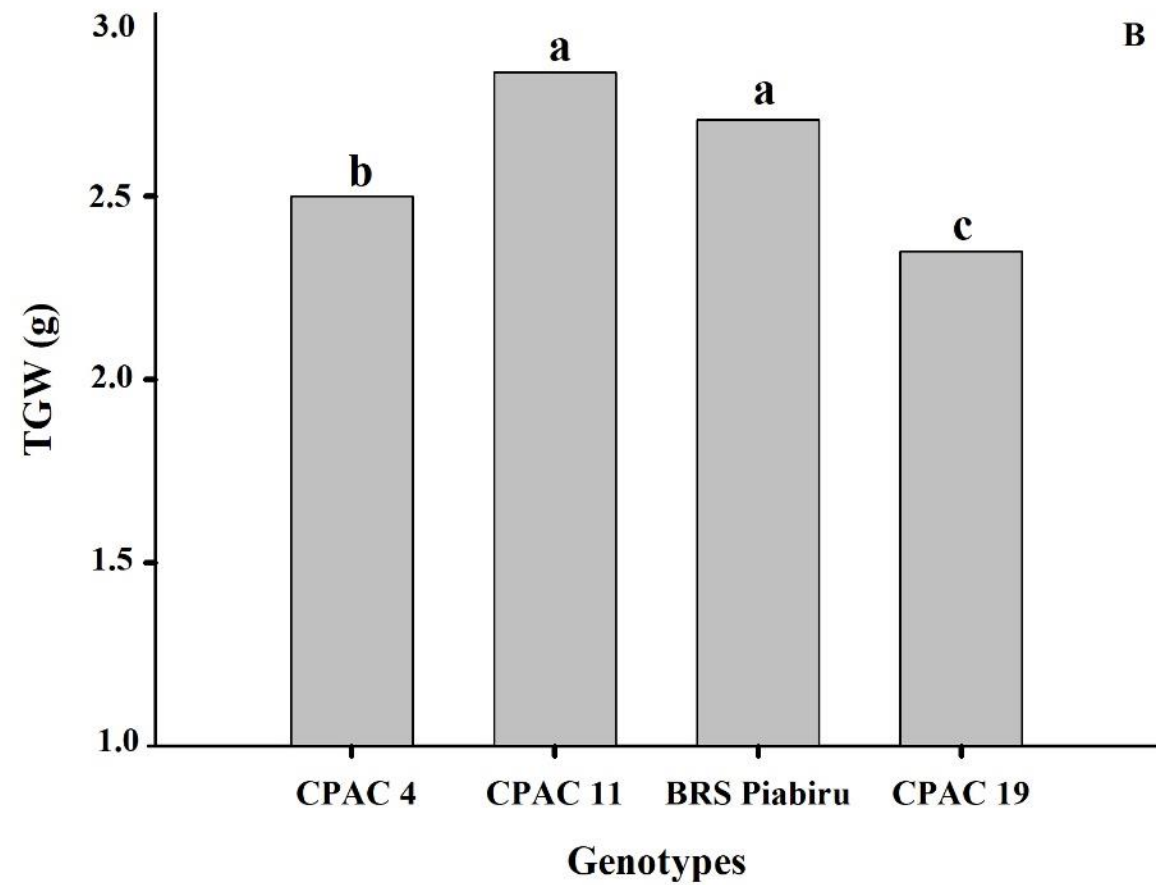

Supplement: Supplementary file 1 [file plants-10-01591-s001.zip › Figura S1.pdf]

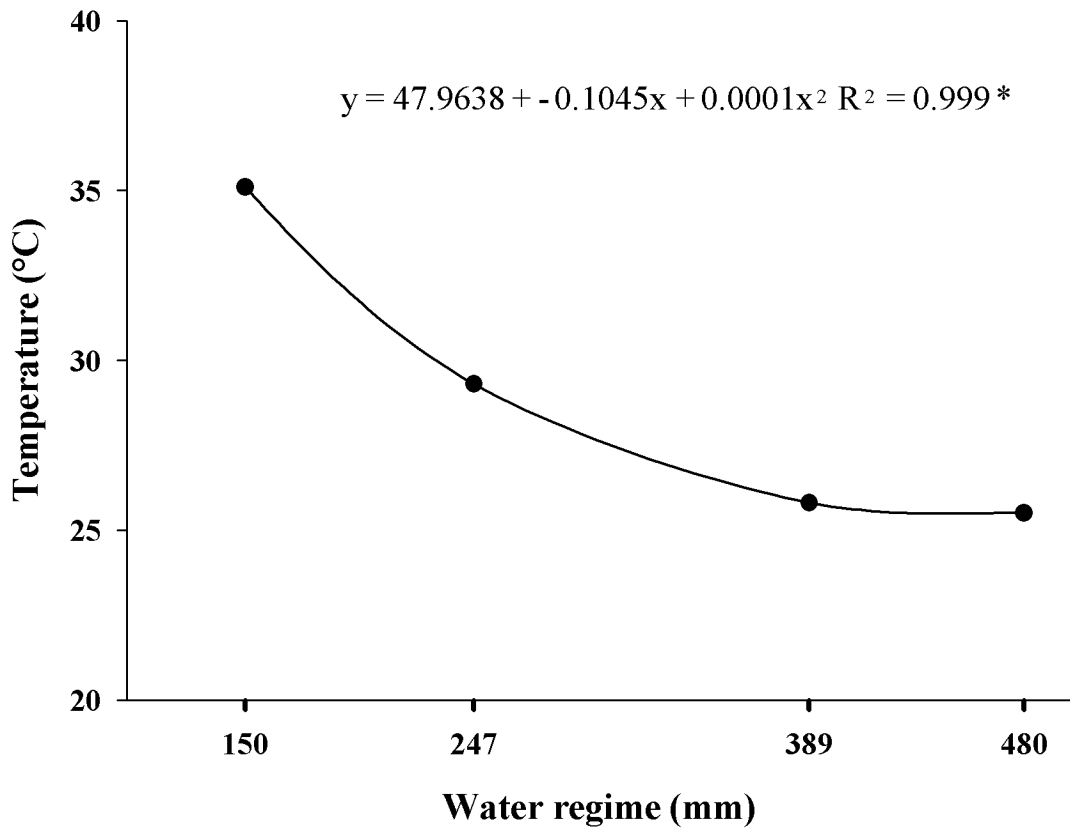

Supplement: Supplementary file 1 [file plants-10-01591-s001.zip › Figure S2.PDF]

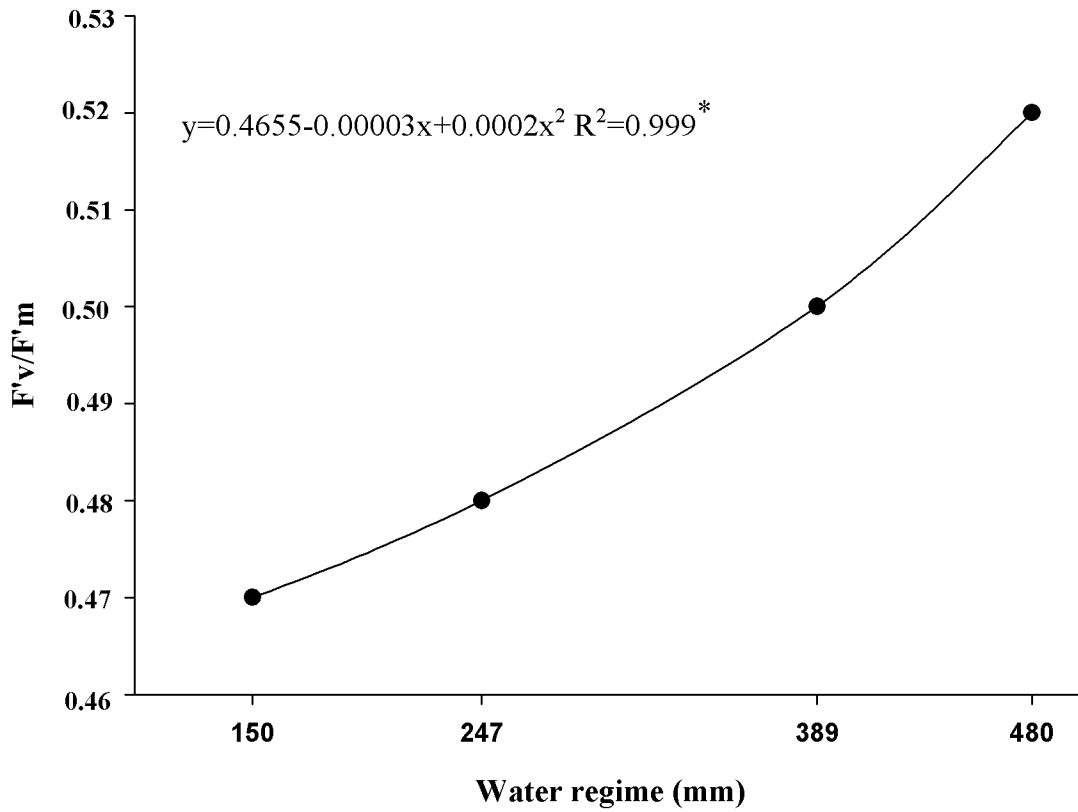

Supplement: Supplementary file 1 [file plants-10-01591-s001.zip › Figure S3.PDF]

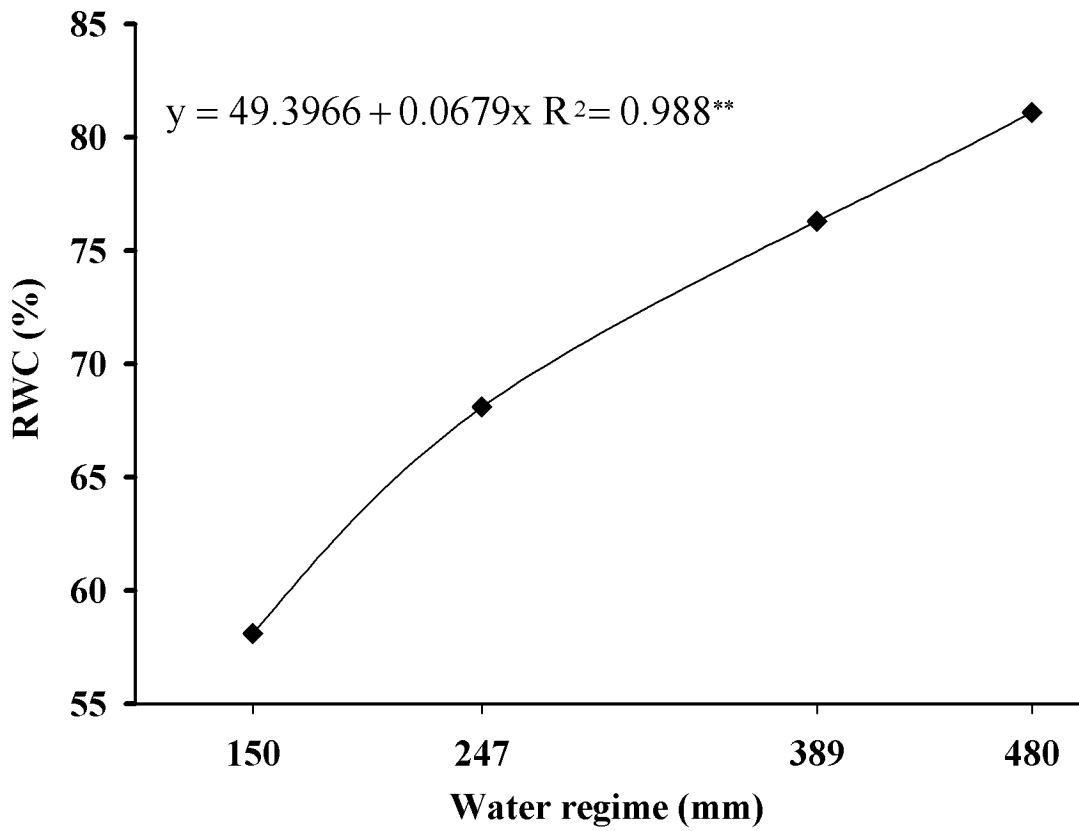

Supplement: Supplementary file 1 [file plants-10-01591-s001.zip › Figure S4.PDF]
